# Quackery and Humbug

**Published:** 1842-04

**Authors:** 


*Quackery and Humbug.—Inflammation.*—The Medical Society of London held its first meeting for the session on the evening of September 27, 1841; Dr. Clutterback, president, in the chair. There was a very full attendance of members and visitors. In some preliminary observations, Dr. Clutterbuck took occasion to allude to several kinds of "quackery" and "humbug" which have lately prevailed, to a certain extent, in the profession. He particularly alluded to some new operations for defective vision, to operations for stammering, and to mesmerism. He classed the three together as being equally worthy of condemnation. He could not help expressing regret and surprise, that such proceedings as those should obtain even a transient notoriety in a profession so strictly one of fact as that of medicine. When, however, they did, unhappily for the public and the profession, succeed in obtaining dupes, then it was the duty of societies, like the Medical Society of London, to expose and condemn the fallacies. This was one great benefit which medical societies conferred upon the public.

After some further observations on the mode of conducting proceedings in the society, a paper was read from the pen of the president, entitled an "Attempt to show that most Diseases originate, directly or indirectly, in Inflammation." The paper was long and elaborate, and we can scarcely venture upon an abstract of it in the present Lancet. Suffice it to say, that by a course of very ingenious reasoning, the author endeavored to show, that almost all diseases, except those which might be considered as merely symptoms, had their origin, more or less remotely, in that state which is denominated inflammation. This inflammation might be of such a character as to be at once recognized, or it might be so insidious as to destroy life, before its presence was detected. Happily, however, the practitioner had, most generally, sufficient evidence of its presence; for, although the four more ordinary signs of inflammation—heat, redness, swelling, and pain—were not to be detected in all cases of inflammation of the vital organs, still, when these were inflamed, there was such an exaltation of the vital properties, so general a febrile state, such an interference with the function of the organ, and such evidence of sympathy manifested by other and remote organs, that the diagnosis was rendered less difficult than it otherwise would be. The author proceeded to enumerate various diseases which originated or were accompanied by inflammation; and concluded by observing, that out of the one hundred and fifty genera enumerated in the nosology of Cullen, one-half of them were merely symptoms or varieties of disease, and not diseases themselves. Of the remaining genera, another half had their origin in inflammation.—*Medical Examiner*, from the *Lancet*.

